# Supplementary material for: Division of labor within psyllids: metagenomics reveals an ancient dual endosymbiosis with metabolic complementarity in the genus Cacopsylla
Source: mSystems. 2023 Sep 28;8(5):e00578-23. doi: 10.1128/msystems.00578-23 (PMC10654072; doi:10.1128/msystems.00578-23)
Supplement: Table S1 — Insect endosymbiont genomes included in the phylogenomics analysis of Ca. Psyllophila symbiotica. [file msystems.00578-23-s0001.docx]

**Supplementary Table S1.** List of insect endosymbiont genomes included in the phylogenomics analysis of ‘*Ca*. Phyllophila symbiotica’.

| **Bacterium** | **Host species** | **Accession** |
| --- | --- | --- |
| *Ca.* Annandia adelgestsuga | *Adelges tsugae* | GCF_003956045.1 |
| *Ca.* Annandia pinicola | *Pineus similis* | GCF_020541245.1 |
| *Ca.* Baumannia cicadellinicola | *Draeculacephala minerva* | GCF_001269425.1 |
| *Ca.* Baumannia cicadellinicola | *Graphocephala atropunctata* | GCF_000754265.1 |
| *Ca.* Baumannia cicadellinicola | *Graphocephala coccinea* | GCF_002855795.1 |
| *Ca.* Baumannia cicadellinicola | *Homalodisca coagulata* | GCF_000013185.1 |
| *Buchnera aphidicola* APS | *Acyrthosiphon pisum* | GCF_000009605.1 |
| *Buchnera aphidicola* | *Aphis craccivore* | GCF_013487785.1 |
| *Buchnera aphidicola* | *Melanaphis sacchari* | GCF_003096055.1 |
| *Buchnera aphidicola* | *Myzus persicae* | GCF_000521585.1 |
| *Buchnera aphidicola* | *Schizaphis graminum* | GCF_000007365.1 |
| Endosymbiont of *Ctenarytaina eucalypti* | *Ctenarytaina eucalypti* | GCF_000287335.1 |
| Endosymbiont of *Heteropsylla cubana* | *Heteropsylla cubana* | GCF_000287355.1 |
| Endosymbiont of *Macroplea mutica* | *Macroplea mutica* | GCF_012571345.1 |
| Endosymbiont of *Plateumaris pusilla* | *Plateumaris pusilla* | GCF_012562765.1 |
| *Ca.* Hamiltonella defensa 5AT | *Acyrthosiphon pisum* | GCF_000021705.1 |
| *Ca.* Hamiltonella defensa MEAM1 | *Bemisia tabaci* | GCF_002285855.1 |
| *Ca.*  Ishikawaella capsulata | *Megacopta punctatissima* | GCF_000828515.1 |
| *Ca.* Mikella endobia | *Paracoccus marginatus* | GCF_900048045.1 |
| *Ca.* Moranella endobia PCIT | *Planococcus citri* | GCF_000219175.1 |
| *Ca.* Moranella endobia PCVAL | *Planococcus citri* | GCF_000364725.1 |
| *Ca.* Nardonella dryophthoridicola Epo | *Euscepes postfasciatus* | GCF_004296535.1 |
| *Ca.* Nardonella dryophthoridicola Pin | *Pachyrhynchus infernalis* | GCF_004296515.1 |
| *Ca.* Nardonella dryophthoridicola NardRF | *Rhynchophorus ferrugineus* | GCF_017656055.1 |
| *Ca.* Nardonella dryophthoridicola Sgi | *Sipalinus gigas* | GCF_004296475.1 |
| *Pseudomonas entomophila* L48 | Pure culture | GCF_000026105.1 |
| *Pseudomonas entomophila* 2014 | Pure culture | GCF_003940785.1 |
| *Pseudomonas entomophila* | Soil microbiome | GCF_018417595 |
| *Ca.*  Purcelliella pentastirinorum | *Oliarus filicicola* | GCF_003391335.1 |
| *Serratia symbiotica* | *Acyrthosiphon pisum* | GCF_008370165.1 |
| *Serratia symbiotica* | *Aphis fabae* | GCF_000821185.2 |
| *Serratia symbiotica* | *Aphis fabae* | GCF_009831665.3 |
| *Sodalis glossinidius* | *Glossina morsitans morsitans* | GCF_000010085.1 |
| *Sodalis* endosymbiont of *Henestaris halophilus* | *Henestaris halophilus* | GCF_900161835.1 |
| *Ca.*  Sodalis pierantonius SOPE | *Sitophilus oryzae* | GCF_000517405.1 |
| *Ca.*  Tachikawaea gelatinosa | *Urostylis westwoodii* | GCF_000828815.1 |
