# Supplementary material for: Division of labor within psyllids: metagenomics reveals an ancient dual endosymbiosis with metabolic complementarity in the genus Cacopsylla
Source: mSystems. 2023 Sep 28;8(5):e00578-23. doi: 10.1128/msystems.00578-23 (PMC10654072; doi:10.1128/msystems.00578-23)
Supplement: Table S2 — Previously published Carsonella genomes used for comparative and phylogenomic analyses. [file msystems.00578-23-s0002.docx]

**Supplementary Table S2.** Table of previously published *Carsonella* genomes used for comparative and phylogenomic analyses.

| **Host species** | **Accession** | **Length (bp)** | **%GC** | **Proteins** | **Pseudogenes** | **rRNAs** | **tRNAs** |
| --- | --- | --- | --- | --- | --- | --- | --- |
| *Bactericera cockerelli* | GCF_002009355.1 | 173802 | 14.80 | 196 | 6 | 3 | 28 |
| *Bactericera trigonica* | GCF_002786955.1 | 174004 | 14.60 | 201 | 0 | 3 | 26 |
| *Ctenarytaina eucalypti* | GCF_000287235.1 | 162589 | 14.00 | 190 | 0 | 2 | 27 |
| *Ctenarytaina spatulata* | GCF_000287255.1 | 162504 | 14.20 | 190 | 0 | 2 | 26 |
| *Diaphorina citri* (China) | GCF_001274515.1 | 174018 | 17.60 | 203 | 4 | 3 | 27 |
| *Diaphorina citri* (Japan) | GCF_000441575.1 | 174014 | 17.60 | 203 | 1 | 3 | 27 |
| *Diaphorina citri* (US) | GCF_013463375.1 | 174118 | 17.80 | 202 | 3 | 3 | 27 |
| *Heteropsylla cubana* | GCF_000287275.1 | 166163 | 14.20 | 192 | 0 | 3 | 28 |
| *Heteropsylla texana* | GCF_000287295.1 | 157543 | 14.60 | 178 | 0 | 2 | 24 |
| *Pachypsylla celtidis* | GCF_000287315.1 | 159923 | 15.60 | 180 | 0 | 2 | 26 |
| *Pachypsylla venusta* | GCF_000010365.1 | 159662 | 16.60 | 182 | 0 | 2 | 26 |
